# Low oxygen levels decrease adaptive immune responses and ameliorate experimental asthma in mice

**DOI:** 10.1111/all.15020

**Published:** 2021-08-01

**Authors:** Mathias Hochgerner, Eva M. Sturm, Diana Schnoegl, Grazyna Kwapiszewska, Horst Olschewski, Leigh M. Marsh

**Affiliations:** ^1^ Ludwig Boltzmann Institute for Lung Vascular Research Graz Austria; ^2^ Division of Pharmacology, Otto Loewi Research Center Medical University of Graz Graz Austria; ^3^ Division of Physiology Otto Loewi Research Center, Medical University of Graz Graz Austria; ^4^ Division of Pulmonology, Department of Internal Medicine Medical University of Graz Graz Austria

**Keywords:** allergy, asthma, dendritic cells, hypoxia, MHC‐II

## Abstract

**Background:**

High‐altitude therapy has been used as add‐on treatment for allergic asthma with considerable success. However, the underlying mechanisms remain unclear. In order to investigate the possible therapeutic effects of high‐altitude therapy on allergic asthma, we utilized a new *in vivo* mouse model.

**Methods:**

Mice were treated with house dust mite (HDM) extract over 4 weeks and co‐exposed to 10% oxygen (Hyp) or room air for the final 2 weeks. Experimental asthma was assessed by airway hyper‐responsiveness, mucus hypersecretion and inflammatory cell recruitment. Isolated immune cells from mouse and allergic patients were stimulated *in vitro* with HDM under Hyp and normoxia in different co‐culture systems to analyse the adaptive immune response.

**Results:**

Compared to HDM‐treated mice in room air, HDM‐treated Hyp‐mice displayed ameliorated mucosal hypersecretion and airway hyper‐responsiveness. The attenuated asthma phenotype was associated with strongly reduced activation of antigen‐presenting cells (APCs), effector cell infiltration and cytokine secretion. *In vitro*, hypoxia almost completely suppressed the HDM‐induced adaptive immune response in both mouse and human immune cells. While hypoxia did not affect effector T‐cell responses per‐se, it interfered with antigen‐presenting cell (APC) differentiation and APC/effector cell crosstalk.

**Conclusions:**

Hypoxia‐induced reduction in the Th2‐response to HDM ameliorates allergic asthma *in vivo*. Hypoxia interferes with APC/T‐cell crosstalk and confers an unresponsive phenotype to APCs.

## Graphical abstract

Hypoxia ameliorates allergic asthma *in vivo*. Hypoxia impairs differentiation of APCs. Hypoxia blocks adaptive immune response by interfering with APC/T‐cell crosstalk.

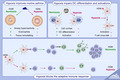

**Abbreviations**
APC: antigen‐presenting cell
HDM: house dust mite

## 1 INTRODUCTION

Asthma is a complex chronic inflammatory disease with a high prevalence.^1^, ^2^ Development and severity of allergic asthma are strongly correlated with exposure to indoor allergens like house dust mite (HDM).^3^, ^4^ There is ample evidence for the beneficial effects of inhaled corticosteroids and other specific anti‐inflammatory treatments for asthma. However, there are side effects that may affect quality of life and there are patients with poor response to pharmacologic treatment.^5^, ^6^, ^7^

High‐altitude climate therapy (HACT) was used before pharmacologic treatments were available and since has still been used to complement pharmaceutical intervention. There are multiple case reports and case series in the literature, describing amelioration of allergic asthma through HACT. A recent meta‐analysis including 21 studies and a total of 907 participants suggested that HACT is an effective therapy for allergic asthma, although there are no randomized controlled double‐blind studies.^8^ In contrast, asthmatic patients from high‐altitude regions, when spending time at lower altitude, experience exacerbation of their disease.^9^ Of note, the positive effect of HACT is still detectable after months of return from high altitude.^10^ Several complementary mechanisms have been postulated to explain these beneficial effects, including reduced allergen load,^11^, ^12^ increased exposure to UV light,^13^ psychosomatic factors^14^ and hypoxia. However, the underlying molecular mechanisms are still poorly understood.

Murine models of asthma allow for investigation of asthma pathogenesis in a controlled environment. Here, we have applied a novel murine HACT model to identify the mechanisms underlying the effects of hypoxia on the asthmatic phenotype. These experiments were complemented by *in vitro* experiments examining the effects of reduced oxygen concentration on the crosstalk between antigen‐presenting cells (APCs) and T‐ and B‐cell responses. Our results indicate that by targeting APC‐effector cell crosstalk or replicating the hypoxic imprinting might serve as a novel therapeutic strategy.

## 2 MATERIAL AND METHODS

### 2.1 Animals

**FIGURE 1:**
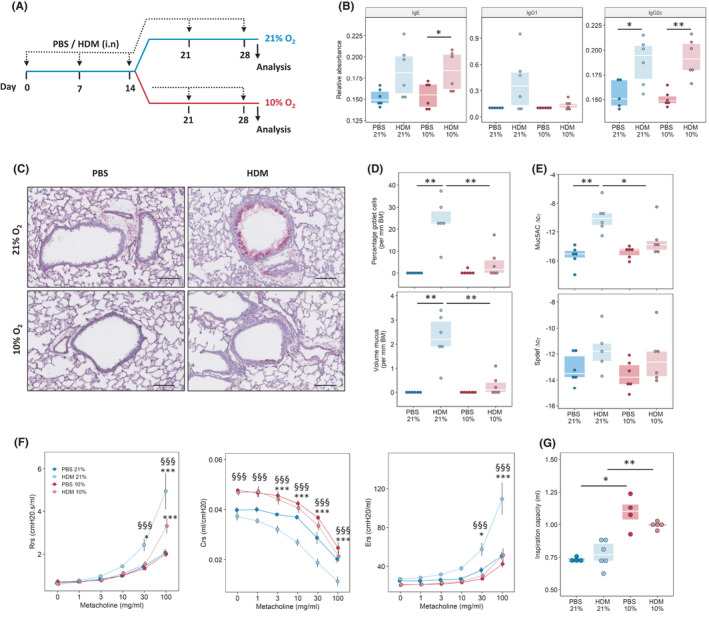
Reduced oxygen concentrations decrease house dust mite (HDM) induced goblet cell hyperplasia and airway hyper‐reactivity. (A) Experimental outline. Mice were sensitized and challenged with a crude extract of HDM or PBS once a week over 4 weeks; after 2 weeks, mice kept under room air (21% oxygen) or reduced oxygen conditions (10% oxygen) for an additional 2 weeks. Analysis was performed 72 h after the last challenge. (B) Serum levels of HDM‐specific antibodies. (C) Periodic acid‐Schiff (PAS) staining of mouse lung tissue isolated from mice exposed to HDM or PBS for 4 weeks with or without the additional exposure to 10% O_2,_ scale bar indicates 100 µm. (D) Quantification of goblet cell hyperplasia and mucus levels, basement membrane (BM). (E) Quantitative PCR analysis of *Muc5AC* and *Spdef* expression in lung tissue. (F) Assessment of airway hyper‐responsiveness and induced by exposure to increasing methacholine (MCh) concentrations; airway resistance (Rrs), compliance (Crs) and elastance (Ers). (G) Inspired capacity (IC) *p < .05, ***p < .001 vs. respective PBS, §§§*p* < .001 vs. HDM (10% O_2_)

C57BL/6J and OT‐II mice were obtained from Charles River (Sulzfeld, Germany). Mice were maintained in isolated ventilated cages with 12 h light/dark cycles. Water and chow were supplied ad libitum. All mouse experiments met EU guidelines 2010/63/EU and were approved by the Federal Ministry of Science, Research and Economics, Vienna, Austria. All measures were taken to keep animal suffering to a minimum. HDM extract was obtained from Greer Laboratories Inc. (Dermatophagoides Pteronyssinus, lyophilized; lot XPB82D3A25), containing: (23.4% total protein, 0.80% DerP1, 0.02% Endotoxin). Mice were treated intra‐nasally with a crude extract of HDM (50 µg protein/25 µl in PBS) once per week for 5 weeks, control mice received PBS. Two groups of mice additionally were subjected to reduced oxygen levels (10% normobaric oxygen) for the last 2 weeks of HDM treatment (Figure 1A). Analysis of lung function parameters and organ collection was performed 72 h after the last challenge.

### 2.2 Assessment of airway hyper‐responsiveness (AHR)

Airway resistance and elastance in response to increased MCh‐challenge were measured via FlexiVent (SciReq Inc., Montreal, PQ, Canada), as previously described,^15^, ^16^ see also Data S1.

### 2.3 Preparation of bronchoalveolar lavage fluid (BALF) and lung tissue

After sacrifice, BALF was obtained by flushing the lung with 1ml PBS 1mM EDTA plus protease inhibitor cocktail (ThermoFisher Scientific, Vienna, AT). Afterwards, lungs were perfused with ice cold PBS and one lobe each was taken for different analyses as described below. For details, see Data S1.

### 2.4 Flow cytometry

Bronchoalveolar lavage fluid and lung single‐cell tissue homogenates (see Data S1) were analysed using a LSRII flow cytometer and analysed with the FACSDiva software (BD Biosciences) or a Beckman Coulter Cytoflex S with CytExpert 2.4, as previously described.^17^ Antibody details and the gating strategy are provided in Table [Link], [Link] in Data S1 and Figures [Link], [Link], respectively. Cells in the BALF are shown as total cell counts, while those in lung tissue were normalized to weight of the lung piece.

### 2.5 Lung immunohistochemistry and quantitative histology

Three µm lung sections were deparaffinized in xylene followed by decreasing concentrations of ethanol and then stained by periodic acid‐Schiff staining (PAS) according to standard protocols. The percentage goblet cells (GC) and mucus volume were quantified using the NewCast software (Visiopharm, Hoersholm, Denmark) as described in the Data S1.^15^, ^18^

Pulmonary vascular muscularization was quantified in sections stained against endothelial cells (rabbit anti‐von Willebrand factor [Dako, Glostrup, Danemark]) and smooth muscle cells (goat anti‐smooth muscle actin [Everest Biotech, Upper Heyford, UK]) as previously described.^19^ Slides were scanned with an Aperio or Olympus slide scanner and analysed using the Visiopharm software. On average 200 ± 90 (mean ± SD) vessels were analysed per slide, the degree of muscularization was calculated as non‐, partially and fully muscularized, depending on coverage of the smooth muscle layer.

### 2.6 Western blotting

Total proteins were isolated from frozen lung samples via mechanically disassociating tissue in liquid nitrogen, then dissolving in RIPA‐buffer. Protein content was measured via Bradford‐assay, adjusted and separated via non‐reducing polyacrylamide‐gels. Proteins were wet‐blotted, blocked with 1% BSA in TBS‐T and detected with anti‐MHC‐II or anti‐alpha tubulin overnight.

### 2.7 RNA isolation and real‐time PCR analysis (qPCR)

RNA isolation from mouse lung was performed via the peqGOLD Total RNA Kit from Peqlab (Erlangen, Germany). Resulting total RNA was transcribed via the iScript™ cDNA Synthesis kit from Bio‐Rad Laboratories (Hercules, CA, USA). Real‐time PCR was performed via the QuantiFast^®^ SYBR^®^ Green PCR kit from Qiagen (Hilden, Germany) on a LightCycler^®^ 480 System from Roche Applied Science (Vienna, Austria). For PCR conditions and analysis, see Data S1. Primer sequences are given in Table [Link], [Link] of the Data S1.

### 2.8 ELISA

Mouse blood was collected via the vena cava, and immunoglobulin levels (IgE, IgG1a and IgG2c) were analysed via ELISA. In brief, 96‐well plates (Maxisorb, Greiner) were incubated overnight with HDM in coating buffer (0.84% NaHCO_3_ in H_2_O, pH 8.3). HDM concentration was 200 µg/ml for IgE, 5 µg/ml for IgG1 and 50 µg/ml for IgG2. The coated plates were incubated with serum samples diluted in PBS 0.1% Tween‐20. HDM‐binding immunoglobulins were labelled with biotin‐tagged anti‐IgE, IgG1 or IgG2 (BD Biosciences, Heidelberg, Germany). Detection occurred via streptavidin‐tagged horse radish peroxidase and BM Blue POD Substrate (Roche), as previously described.^17^

In mouse BALF (collected as above) and lung homogenate, IL‐4, IL‐13 and IFNγ were measured via Ready‐SET‐Go ELISA kits from Ebioscience/ThermoFisher (Vienna, Austria).

Specific and total IgE antibody levels in serum of human blood donors were measured by ImmunoCap 250 (Thermo Fisher Scientific, Waltham, USA) according to the manufacturer's instructions.

Supernatants from cell culture experiments were diluted 1:1 in assay buffer and analysed via Biolegend LegendPlex immunoassay according to the manufacturer's instructions (LegendPlex mix and match mouse panel).

### 2.9 Mouse in vitro assays

Mouse splenocytes were isolated and enriched for immune cells with Pluriselect Lympho Spin Medium. In brief, spleens were mechanically disassociated into a cell suspension, which was overlaid on PBMC spin medium. After centrifugation for 30 min, 800 g, 25°C, the interphases were collected. 2 × 10^5^ enriched splenocytes were plated in 200 µl medium (PRMI 10% FBS, 1% Pen/Strep and 1% Glutamine) in 96‐well plates and stimulated with 100 µg/ml of HDM plus 20 ng/ml soluble anti‐CD28 (BioLegend) or 4 µl/well anti‐CD3‐CD28 beads (aCD3/28) respectively (ThermoFischer Scientific). Cells were incubated at standard conditions (37°C, 5% CO_2_) under 21% O_2_ or 1% O_2_. Cells were harvested at day 8, and proliferation of adaptive effector cells was analysed via flow cytometry.

Bone marrow (BM) was flushed from mouse femur and tibia, disassociated, and 2 × 10^6^ cells per well were plated in 24‐well plates in 1.5 ml differentiation medium (medium as above plus 1% non‐essential amino acids, 1% Sodium Pyruvate and 20 ng/ml GM‐CSF from PeproTech). At day 3, differentiation medium was changed and non‐adherent cells were discarded. At day 6, BM‐ antigen‐presenting cells (APCs) were stimulated with 100 µg/ml HDM for 7 h. T cells were isolated from homogenized mouse spleens via Biolegend Mojo Sort^TM^ Mouse CD4 T Cell Isolation Kit, stained with carboxyfluorescein succinimidyl ester (CFSE; 10 µM, 10 min, 37°C) and co‐cultured (TCs:DCs =1:10) with HDM‐stimulated BM‐APCs (as above) and 20 ng/ml soluble anti‐mouse CD28 for 7 days.

For OT‐II co‐culture, BM was harvested from OT‐II‐mice and differentiated as above under 21% O_2_ or 1% O_2_. BM‐APCs were stimulated with 1 µg/ml Ovalbumin‐peptide 323–339 (Sigma‐Aldrich O1641‐1MG) for 7 h under 21% O_2_ or 1% O_2_ and then co‐cultured (TCs:DCs =1:10, 1:20 or 1:50) with OT‐II T cells (isolated as above, without CFSE‐staining) under 21% O_2_ or 1% O_2_ for 7 days.

### 2.10 Human in vitro assays

Peripheral blood was collected by venipuncture from 7 donors with self‐reported HDM‐allergy and confirmed HDM‐specific IgE, as well as 8 healthy controls according to a protocol approved by the Ethics Committee of the Medical University of Graz (17–291 ex 05/06). Control blood donors reported no allergy‐related complaints (details are included in Table [Link], [Link]). Written and informed consent was obtained from all patients included in this study. Oral cortical steroid (OCS) users were excluded, and inhaled cortical steroid (ICS) use was abstained for 24 h prior to blood sampling. In brief, blood cells and plasma were separated by centrifugation, erythrocytes were removed by dextran sedimentation and PBMC (buffy coat) were separated from PMNLs (pellet) by density gradient centrifugation using Histopaque 1077.^20^ PBMCs were treated like mouse splenocytes, except HDM was used at 25, 50 and 100 µg/ml and cells incubated for 2, 5 or 8 days, as indicated.

### 2.11 Statistics

Data are presented as individual data points and box and Tukey whisker plots or mean +/‐ SEM using R statistical environment (Version 3.6.1). Flow cytometry data are additional presented as via heat map, using the pheatmap package in the R. Data were log_10(x+1)_ transformed and Z‐scaled according to the cell type and samples clustered according to Euclidean distances. Statistical differences between the groups were determined by the Kruskal‐Wallis with Dunn's post hoc test or one‐way ANOVA with Tukey's post hoc test as appropriate. For the comparison of airway hyper‐responsiveness, a two‐way ANOVA with Bonferroni's post hoc test was used. Statistical significance is indicated in the figures as follows; *p < .05, **p < .01, ***p < .001.

## 3 RESULTS

### 3.1 Hypoxia decreases airway remodelling and hyper‐responsiveness

In order to investigate the direct effects of hypoxia on the progression of experimental asthma, mice were first sensitized and challenged with HDM, then randomly divided into two groups, one at room air (21%) and one at 10% O_2_ (Figure 1A). HDM sensitization was confirmed by the presence of HDM‐specific immunoglobulins in the serum (Figure 1B). As expected, 2 weeks of exposure to 10% O_2_ resulted in mild signs of pulmonary hypertension, as shown by increased right heart hypertrophy and pulmonary vascular remodelling (Figures [Link], [Link]A–C). Generally, mice under 10% O_2_ were lighter than controls (Figures [Link], [Link]D), which was due to decreased weight gain and not due to weight loss (data not shown). HDM‐treated mice at 21% O_2_ showed high numbers of goblet cells and mucus levels while in HDM‐10% mice these numbers were significantly reduced (Figure 1C,D). In line with this observation, *Muc5AC* gene expression in HDM‐10% treated mice was lower than the room air controls. While expression of the goblet cell differentiation factor *Spdef* was slightly decreased at 10% O_2,_ the difference to 21% O_2_ was not significant (Figure 1E). Analysis of lung function revealed pronounced AHR in normoxic HDM‐treated mice while Hyp‐HDM mice exhibited significantly less AHR, as shown by attenuated airway resistance, compliance and elastance (Figure 1F). Interestingly, Hyp‐mice exhibited increased inspiratory capacity (Figure 1G), which is indicative of reduced air trapping. There was a significant interaction between HDM treatment and Hypoxia (*p* for interaction <.001), suggesting that hypoxia specifically inhibited the asthmatic response to HDM.

### 3.2 Hypoxia reduces inflammation upon HDM treatment

**FIGURE 2:**
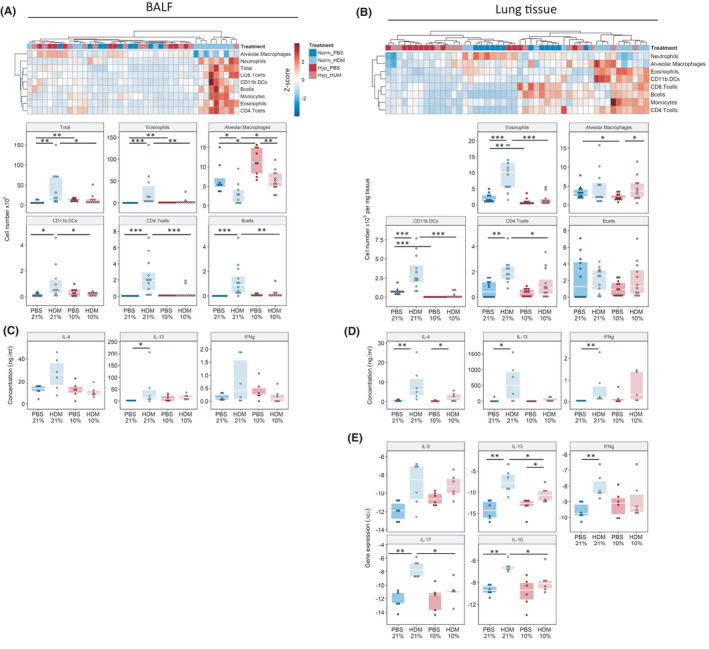
Exposure to low oxygen conditions attenuates the HDM‐induced immune response. Flow cytometry analysis of inflammatory cell content in the bronchiolar alveolar lavage fluid (BALF), (left panels) and lung tissue (right panels). (AB) Heat map representation of all analysed inflammatory cell populations using log_10+1_ transformed values, cell populations are z‐scaled with red indicating highest abundance and blue lowest. Total cell counts (BALF) and cell counts normalized to lung weight (Lung tissue) are also represented by dotplot with boxplot overlays. Analysis of inflammatory cytokines in (C) BALF and (D) lung tissue via ELISA and via (E) qPCR in the lung. Gating strategy in Figures [Link], [Link]. *p < .05, **p < .01, ***p < .001

We next compared the pulmonary inflammatory environment in all groups. Inflammatory cells were identified using flow cytometry (gating strategy in Figures [Link], [Link]) and visually represented via heat maps, which revealed a strong similarity of the inflammatory profiles of 21% O_2_‐HDM mice (Figure 2A,B upper panels). The BALF from 21% O_2_‐HDM mice showed a significant increase in total cells, primarily consisting of eosinophils and CD11b^+^ dendritic cells, CD4^+^ and CD8^+^ T cells and B cells (Figure 2A). This inflammatory cell influx was mostly abrogated under low oxygen conditions. Interestingly, PBS‐10% mice displayed slightly increased amounts of alveolar macrophages compared to their normoxic controls. Flow cytometric analysis of lung tissue showed a similar trend, with HDM‐treated mice showing increased amounts of immune cells compared to PBS‐treated controls and a significant reduction in eosinophilic recruitment under hypoxia (Figure 2B). Full gating strategy given in Figures [Link], [Link]. Analysis of cytokine levels in BALF and lung tissue revealed increased levels of the Th2 cytokines IL‐4 and IL‐13 in response to HDM treatment (Figure 2C,D). Again, exposure to reduced O_2_ concentrations abrogated the effects of HDM, causing no detectable increase in these cytokines. Cytokine ELISA measurements were confirmed via qPCR of lung tissue, which additionally revealed an increase in IFNγ, IL‐10 and IL‐17 upon HDM‐stimulation, which again was almost completely suppressed in HDM‐10% mice (Figure 2E).

### 3.3 Induction of antigen‐specific adaptive immune response *in vitro* is critically dependent on oxygen

**FIGURE 3:**
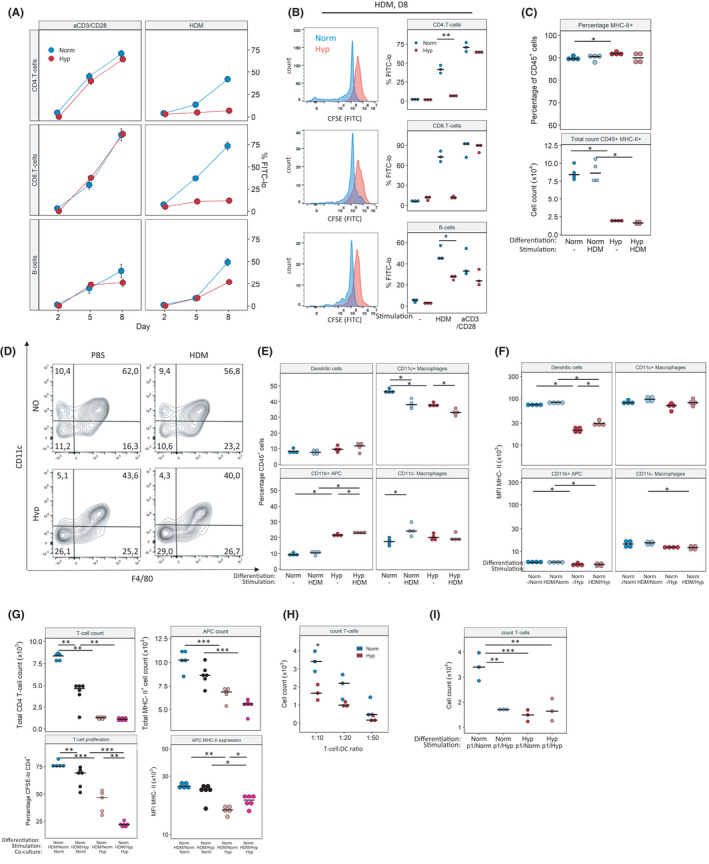
Hypoxia impairs the HDM‐induced adaptive immune response. (A) Mouse splenocytes were stimulated *in vitro* with 100 µg/ml HDM or aCD3/CD28‐beads for the indicated number of days under normoxia (Norm) or hypoxia (Hyp). Flow cytometry analysis of T‐ and B‐cell proliferation as % CFSE‐lo of parent population. (B) Example flow cytometry overlay of cell proliferative response as measured by CFSE dilution (left). Quantification of cells from (A) on day 8 (right). Antigen‐presenting cells (APCs) were differentiated from mouse bone marrow to BM‐APCs for 6 days under Norm or Hyp, then stimulated for 7 h with 100 µg HDM under the same conditions. (C) Relative and absolute quantification of CD45+MHCII+cells. (D, E) Identification and quantification of differentiation under Norm or Hyp, gated on CD45^+^MHCII^+^CD11b^+^. (F) BM‐APCs were generated under Norm, stimulated under Norm or Hyp (F). (G) CD4^+^ T cells were isolated from mouse spleen and co‐cultivated with HDM‐stimulated BM‐APCs for 7 days. APC differentiation Norm, HDM‐stimulation Norm or Hyp, APC/T‐cell co‐culture Norm or Hyp as indicated. (H) BM‐APC from OT‐II mice was co‐culture under Norm or Hyp with CD4^+^ T cells at ratios 10:1, 20:1 and 50:1 for 7 days in the presence of Ovalbumin‐peptide (p1) 323–339, and the number of T cells and live cells quantified. (I) Reciprocal co‐culture experiments performed with BM‐APC differentiated under Norm or Hyp, pulsed with 1 µg/ml peptide and incubated with isolated T cells at under Norm or Hyp. Full gating strategy is given in Figures [Link], [Link]. Mean fluorescence intensity (MFI), *p < .05, **p < .01, ***p < .001

In order to examine the reasons for dampened immune response, we performed a series of *in vitro* experiments. Splenocytes were isolated from treatment‐naive mice and stimulated *in vitro* with HDM under normoxic (Norm) or hypoxic (Hyp) conditions. Under Norm, HDM induced clonal expansion of CD4^+^ T cells, CD8^+^ T cells and B cells, while under Hyp none of these populations showed any reaction to HDM (Figure 3A,B). Interestingly, mouse splenocytes stimulated with aCD3/CD28‐beads were unaffected by hypoxia, indicating that polyclonal T‐cell response upon direct activation was not affected. Based on this data, we hypothesized that hypoxia did not impair the function of the effector cells but rather interfered with the function of the antigen‐presenting cells (APCs).

We therefore generated mouse bone marrow‐derived APCs for further experiments. Surprisingly, the generation of BM‐APCs was also affected by hypoxia. Compared to Norm, BM precursors differentiated under Hyp yielded significantly lower amounts of CD45^+^MHC‐II^+^ cells (Figure 3C). Furthermore, hypoxia BM promoted the differentiation of more CD11b^+^ APC (CD11b^+^ CD11c^−^ F4/80^−^) cells at the expense of CD11c^+^ macrophages (CD11b^+^ CD11c^+^ F4/80^+^) (Figure 3D,E). Next, we stimulated BM‐APCs with HDM and analysed activation status via flow cytometry. APCs generated under Norm showed strongly reduced MHC‐II levels when exposed to hypoxia, both with and without HDM‐stimulation (Figure 3F), while CD11b^+^CD11c^+^ DCs generated under Hyp displayed increased MHC‐II expression when transferred to Norm, again both in stimulated and in unstimulated conditions. Other BM‐APC‐subsets showed no significant reaction to re‐oxygenation (Figures [Link], [Link]B). Full gating strategies given in Figures [Link], [Link].

To determine how a hypoxic environment can alter the interaction of APC and T cells, we isolated CD4^+^ T cells from mouse spleens. Normoxically generated BM‐APCs were stimulated with HDM under Norm or Hyp, then co‐cultivated with T cells again under Norm or Hyp for 8 days (Figure 3H). Cells which were both stimulated and co‐cultivated under Norm generated a robust T‐cell response; stimulation and co‐culture under Hyp led to impaired T‐cell proliferation. When Hyp‐stimulated APCs were co‐cultivated under Norm, T cells still respond, albeit in a reduced manner. However, when APCs and T cells were co‐cultivated under Hyp, the T‐cell expansion was almost completely abrogated. In these conditions, oxygen level at APC stimulation did not influence T‐cell numbers (Figure 3H). This suggests that APC‐T‐cell‐crosstalk is oxygen‐dependent. Oxygen conditions during APC stimulation also seem important, but to a lesser degree.

In order to confirm our findings in another model, we repeated the experiment with OT‐II cells: BM from OT‐II mice was harvested and differentiated into APCs as before, then stimulated with ovalbumin‐peptide and finally co‐cultivated with OT‐II CD4+ T cells. Similar to above, APC stimulation and APC/T‐cell co‐culture were performed under Norm and Hyp and revealed decreased T‐cell numbers when cultured under Hyp (Figure 3H). Additionally, this time we also performed BM‐differentiation under both Norm and Hyp. Under Norm‐differentiated APCs stimulated under Norm induced a robust response from OT‐II T cells. Similar to the HDM in vitro model, the T‐cell response was severely impaired when APC/T‐cell co‐cultivation was performed under Hyp. Interestingly, when APCs were either generated under Hyp or pulsed/co‐cultured under Hyp, the T‐cell response was strongly ablated (Figure 3I). Examination of MHC‐II expression at day 8 of co‐culture revealed a small increase in MHC‐II MFI in CD11c^+^, when cells were co‐cultured under hypoxia (Figures [Link], [Link]D).

### 3.4 Up‐regulation of MHC‐II in APCs of the lung is impaired by hypoxia

**FIGURE 4:**
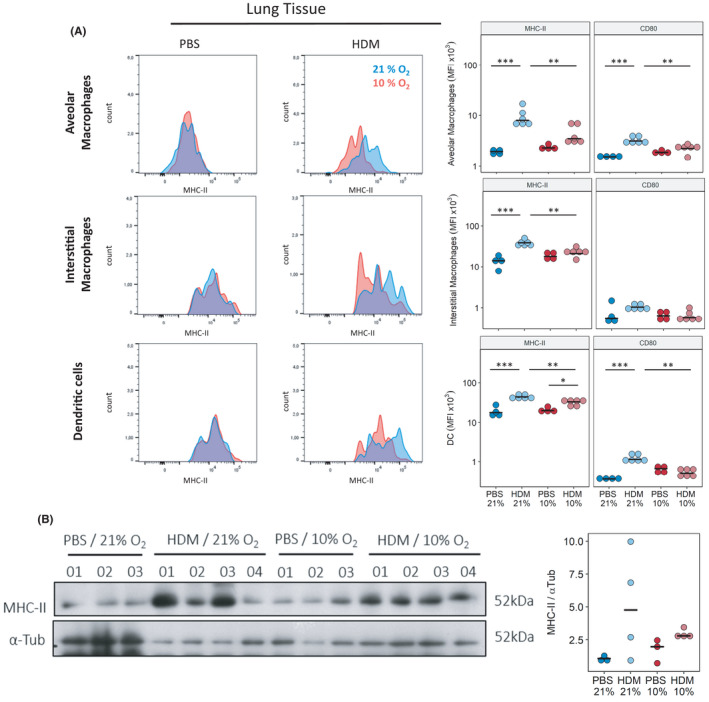
Exposure to reduced oxygen concentrations in vivo reduces MHC‐II levels in lung APCs in response to HDM challenge. Lung tissue was harvested from mice treated with PBS or HDM for 4 weeks and co‐exposed to hypoxia (Hyp) or kept at normoxia (Norm) for the last 2 weeks. (A) Flow cytometry analysis of MHCII‐expression in different APC‐populations in lung tissue. (B) Quantification of (A) and of CD80‐expression. (C) Western blot analysis of total MHC‐II levels in lung tissue, α‐Tub represents loading control. (D) Quantification of (C). Full gating strategy is given Figures [Link], [Link]. *n* = 4–6, representative of 2 independent experiments *p < .05, ***p < .001

In order to verify our *in vitro* observations, we returned to our *in vivo* HDM model using flow cytometry to quantify expression of MHC‐II and CD80 in several APC‐populations within the lung. Residual alveolar macrophages (CD11b^low^ CD11c^+^ Siglec‐F^+^), interstitial macrophages (CD11b^+^ CD11c^low^, CD64^+^ Siglec‐F^−^) and dendritic cells (CD11b^+^ CD11c^+^ CD24^+^ MHC‐II^high^) all exhibited strong upregulation of MHC‐II and co‐receptors in response to HDM under normoxia. This upregulation was impaired under hypoxia in all observed populations (Figure 4A). Gating strategy given in Figures [Link], [Link]. HDM‐induced upregulation of MHC‐II and its impairment under hypoxia were also confirmed via Western blot of total lung tissue (Figure 4B).

### 3.5 Human adaptive immune response is dependent on oxygen level

**FIGURE 5:**
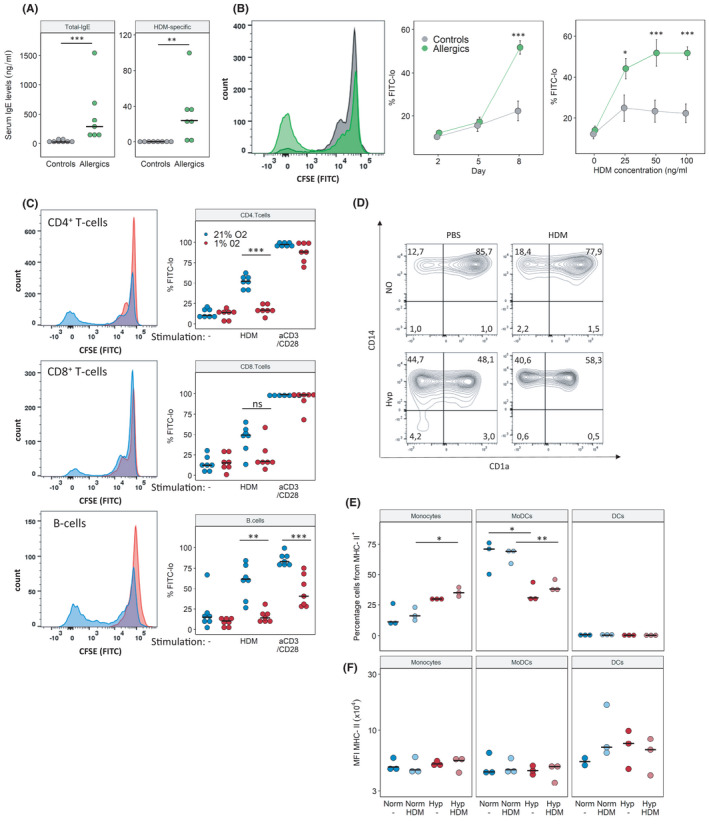
The human adaptive immune response to HDM is impaired under hypoxic conditions. Immune cells were isolated from peripheral blood of HDM‐allergic patients or healthy donors. PBMCs were stimulated in vitro with 25, 50 or 100 µg HDM or aCD3/28 beads under normoxia (Norm) and hypoxia (Hyp) for 8 days. (A) Total IgE and HDM‐specific IgE measured by ELISA from serum of blood donors. (B) CD4^+^ T‐cell proliferation in response to HDM stimulation was measured by flow cytometry (non‐allergic vs. allergic subjects, Norm only). Representative CFSE histogram (left), proliferation response over time (middle), proliferation with increasing HDM concentrations at day 8 post‐stimulation (right). (C) Flow cytometry analysis of cell proliferation on day 8 as % CFSE‐lo of T and B cells, either unstimulated (‐), in response to 100 µg/ml HDM or aCD3/CD28 stimulation. (D) Flow cytometry analysis of antigen‐presenting cells (APCs) on day 8 as % of CD45^+^MHCII^+^. Monocytes (CD14^+^CD11b^+^CD1a^−^), MoDCs (monocyte‐derived dendritic cells, CD14^+^CD11b^+^CD1a^+^), DCs (dendritic cells, CD1a^+^CD11b^+^CD14^−^). (E) Quantification of subpopulations of APCs stimulated with or without HDM under Norm or Hyp. (F) Mean fluorescent intensity (MFI) of MHCII in populations from (E). Full gating strategy is given Figures [Link], [Link]. *n* = 7 (A–C), *n* = 3 (D–F) *p* < .05, **p < .01

In order to confirm our findings in a human system, we repeated our *in vitro* experiments with human PBMCs collected from HDM‐allergic and non‐allergic donors. Total IgE and HDM‐specific IgE levels are shown in Figure 5A. As expected, PBMCs from allergic humans showed a robust adaptive immune response to HDM. PBMCs from non‐allergic donors also reacted to HDM, but to a far lesser extent (Figure 5B). In line with our mouse data, human adaptive immune (both T‐ and B‐cell) response to HDM was strongly suppressed by hypoxia (Figure 5C). Again, the polyclonal T‐cell response to direct stimulation via aCD3/CD28‐beads was unaffected. Interestingly, the B‐cell response upon aCD3/28 was also reduced under hypoxia; since aCD3/28 acts on the T cells, which in turn activate B cells, again suggesting that hypoxia interferes with cell‐cell communication.

Similar to our observations in mice, human APCs displayed changes in differentiation upon hypoxia: the bulk of CD45^+^MHC‐II^+^ cells were CD14^+^ monocytes and CD14^+^CD1a^+^ monocyte‐derived dendritic cells (MoDCs). In hypoxic co‐cultures, MoDCs were reduced in favour of monocytes, indicating that hypoxia impairs monocyte differentiation. In HDM‐stimulated co‐cultures, MHCII+B cells were not significantly changed; however, their amount was increased in unstimulated hypoxic samples. MHCII+CD4+ T cells were detected in low numbers; in normoxic co‐cultures, HDM‐stimulation led to a significant decrease of these cells, while under Hyp this difference was not significant (Figures [Link], [Link]C). The expression of MHC‐II in these cell populations was not significantly changed. Gating strategies given in Figures [Link], [Link]A,B.

## 4 DISCUSSION

Since the 1950s, there have been reports on successful high‐altitude climate therapy (HACT); interestingly, the beneficial effects of HACT started a few days after arrival in the clinic but often persisted for up to half a year after return to low level.^21^, ^22^ Although pharmacological treatment of asthma is highly effective, there are some side effects, there are non‐responders, and the effects are not sustained after withdrawal of the drug.

We here introduce a novel mouse model which recapitulate several effects of HACT in allergic asthma, and allow for understanding some of the underlying molecular and cellular mechanisms. In our model, lower concentrations of oxygen lead to a functional improvement in several important hallmarks of asthma, namely AHR, goblet cell hyperplasia and airway inflammation. Analogous to observations of allergic patients undergoing HACT,^23^ hypoxic HDM‐mice show reduced eosinophilia and Th2 cytokines. Our results support previous studies, where similar effects were observed in asthma patients when moved from lower altitude to high altitude.^23^, ^24^ Historically, three main hypotheses were held as to the mechanism underlying HACT: Allergen avoidance,^11^ increased UV‐radiation^24^ or avoidance of psychosomatic triggers.^14^ Our data suggest that reduced oxygen concentration is a very important factor explaining at least some of the beneficial effects of HACT. Hypoxia has been investigated in the context of asthma before; one study found intermittent hypoxia to aggravate asthma,^25^ while another study reported the opposite.^26^ Older experimental models used intermittent hypoxia, resembling conditions that occur in patients in sleep apnoea. In contrast to previous studies, our model used sustained hypoxia, resembling the situation of high‐altitude therapy.

However, it should be noted that our study uses severe hypoxia, which differs from the mild hypoxia that patients would experience in a HACT‐clinic. Nevertheless, our study identifies several principle hypoxia‐driven molecular pathways that may play a role in HACT.

Under hypoxic conditions, T‐helper cells remain functional.^27^ Our *in vitro* findings confirm this by showing that direct activation of CD4^+^ T cells is unaffected by hypoxia. However, hypoxia does impair specific allergen‐induced T‐cell responses both *in vitro* and *in vivo*, suggesting that hypoxia is not so much affecting adaptive effector cells but their activation by APCs. Based on our in *vitro data*, we hypothesize that hypoxia interferes with APC/T‐cell crosstalk.

While previous studies speculated about the role of APCs in the conveyance of the positive effect of HACT, most studies have only used peripheral blood samples to explain the effects.^24^, ^28^ In recent years, however, we have learned that there are striking differences between circulating and tissue‐resident immune cells.^29^ Therefore, we must investigate the actual immune status of the lung. Accordingly, we aimed to investigate the functional consequences of HACT *in situ* and describe the cellular and molecular changes leading to this therapeutic effect.

Within the lung, APCs can be categorized into macrophages and dendritic cells (DCs). While similar, these cell types differ in origin and function. DCs are the most potent APCs and professional sentries of the immune system. However, relatively little is known how reduced oxygen concentrations affect these cells. The effects of hypoxia on macrophages have been investigated by several groups, but the results are contradictory. Some investigations have shown that hypoxia increases phagocytosis,^30^ others have shown the opposite.^31^ The same is true for M‐1 polarization.^32^, ^33^ One cause for the conflicting results may be the diversity of macrophages and the fact that different cells react differently to hypoxia. Only recently, scientists have begun to appreciate the difference of embryonic, self‐renewing macrophages and macrophages derived from bone marrow precursors (reviewed in^34^). In an *in vivo* setting, as well as in patients, a significant part of APCs reacting to antigen and/or hypoxia will be of the embryonic macrophage lineage.

Macrophages have been demonstrated to react to changes in oxygen levels with a multitude of molecular responses. Several *in vivo* studies have demonstrated the importance of hypoxic macrophages. In a mouse model of rheumatoid arthritis, myeloid knock‐out of Hif1α signalling has a potent anti‐inflammatory effect^35^; however, it also renders mice susceptible towards bacterial inflammation and impairs wound healing.^36^ The current state of knowledge depicts oxygen conditions as a key regulator of macrophage function, but how exactly hypoxia affects macrophages, and how this in turn affects the outcome of inflammatory disease, is strongly dependent on the context. Much more research is needed to decipher the complex interplay of macrophage subtype, mode of activation, oxygen levels and additional environmental influences such as cytokines, nutrients, mechanical pressure or others.

Our *in vivo* data show that lung APCs are strongly affected by hypoxia, failing to upregulate MHC‐II and CD80 upon HDM challenge. Furthermore, supporting previous findings,^37^ our data show that hypoxia affects the development of APCs from BM precursors, both reducing total amount but also causing a shift in generated subsets of BM‐APCs. Finally, we could show that APCs generated under hypoxia fail to initiate a T‐cell response, irrespectively of oxygen conditions under stimulation or T‐cell‐co‐culture. Therefore, we hypothesize that hypoxia during differentiation of APCs causes a ‘hypoxic imprinting’, and thereby a lasting reduction in their ability to initiate adaptive immune responses. The exact genetic or epigenetic mechanisms governing this imprinting will still need to be discovered. Our observations might offer an explanation as to why the positive effect of HACT persists after the patient has returned from the high‐altitude stay.

Even under steady‐state conditions, APCs permanently leave their original tissue to migrate to the next draining lymph node. Under inflammatory conditions, this migration is massively accelerated. As a consequence, the tissue is re‐populated with APCs; this happens partially through multiplication of remaining APCs *in situ*, partially through immigration and differentiation of blood monocytes/BM‐derived precursor cells (reviewed in^38^). We propose that a stay at lower oxygen conditions alters the differentiation of re‐populating APCs and therefore changes composition and function of the tissue‐resident APCs. This, in turn, results in an altered immune response when the tissue is again exposed to antigen. After exposure to higher oxygen, these ‘hypoxically imprinted tissue APCs’ would persist for weeks or months, until finally again being replaced by normoxically generated APCs.

The immune system is incredibly complex; its abilities are dependent on a multitude of cell types to differentiate and interact with each other, as well as with the surrounding tissue and eventual pathogens. Therefore, an immune response, particularly one underlying a pathological condition, can only be understood in an *in vivo* setting. We suggest our model for further investigations of hypoxia effects on allergic asthma. Especially the ‘hypoxic imprinting’ of APCs needs to be studied in further detail and *in vivo*. Further research into the hypoxia‐mediated effects on the immune cells and their crosstalk may lead to durable effects on allergic asthma that can be attained by small interfering molecules or biologicals.

Our study has some limitations: First, our principle findings are based upon mouse experiments; while our data very well recapitulated the human situation, some details are not directly translatable. The perfect way to test translatability would be lung biopsies from patients before, at the end and sometime after HACT to study APCs in the tissue. Obtaining such samples, however, will be a challenge for itself.

Next, our *in vivo* experiments were performed at 10% oxygen; outside of a laboratory, these conditions are found at altitudes of 5500 metres. However, most patients undergoing HACT reside at 2500 metres altitude or below. However, our study provides excellent proof of concept new data, a novel hypothesis and possible molecular mechanisms to investigate.

Numerous studies have described a positive effect of HACT on humans; however, altitude of the treatment facilities and duration spent there varies. Whether a longer stay at moderate altitude confers benefits comparable to a stay at higher altitudes and what is the minimum time and altitude to robustly confer a longer lasting effect still needs to be determined.

This paper introduces an *in vivo* model to investigate the impact of hypoxia on adaptive immunity; we hope that similar protocols as ours will be of use to further unravel the molecular mechanisms governing this immune‐dampening effect. We propose to use our model to further dissect monocyte‐to‐APC differentiation and cell migration/retention in the lung. Further research in this direction is necessary to deepen our understanding, but hardly possible in humans. Ultimately, we hope that our work contributes to a deeper understanding of adaptive immune modulation and possibly reveals new therapeutic targets and treatment strategies for immune driven diseases.

## CONFLICT OF INTEREST

The authors declare no competing financial interests.

## Supporting information

Fig S1

Fig S2

Fig S3

Fig S4

Fig S5

Tab S1

Tab S2

Tab S3

Data S1
