# Supplementary figures and images for: Low oxygen levels decrease adaptive immune responses and ameliorate experimental asthma in mice
Source: Allergy. 2021 Aug 1;77(3):870–82. doi: 10.1111/all.15020 (PMC9290649; doi:10.1111/all.15020)

Supplementary Figure E1

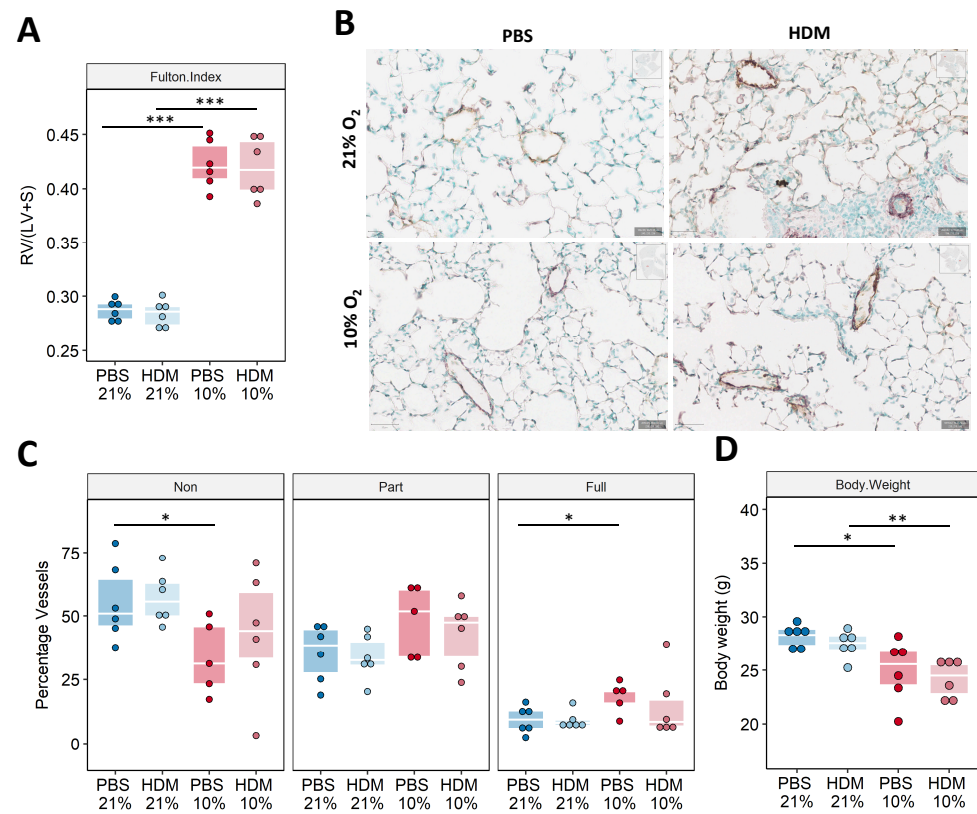

Supplement: Supplementary file 1 — Fig S1 [file ALL-77-870-s005.pdf]

Supplementary Figure E2

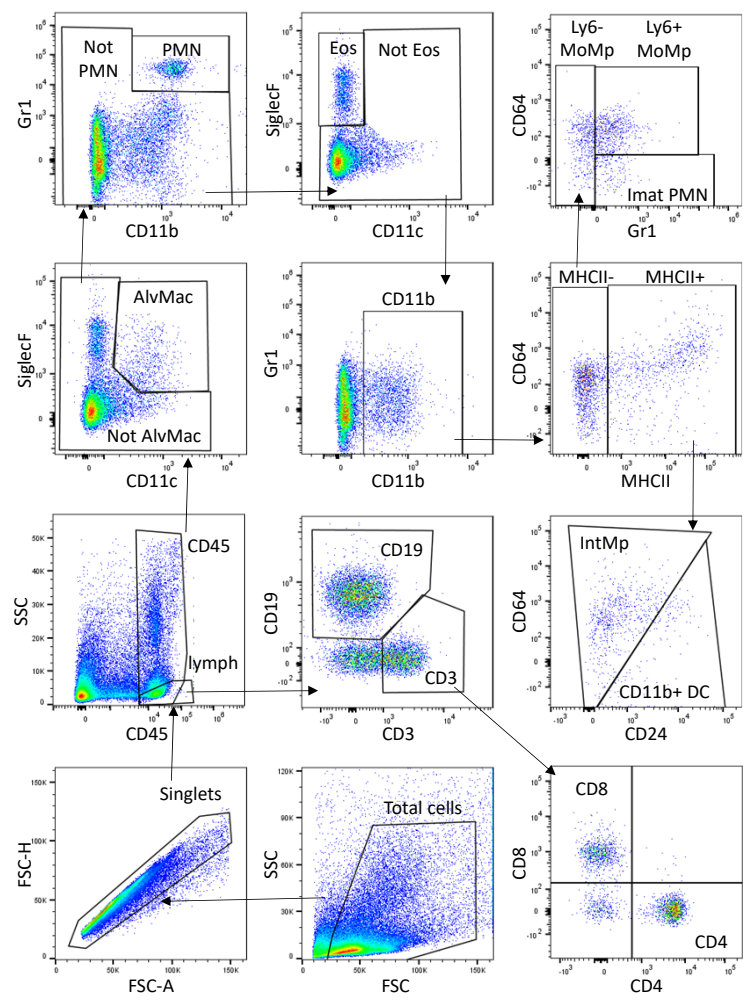

Supplement: Supplementary file 2 — Fig S2 [file ALL-77-870-s008.pdf]

Supplementary Figure E3

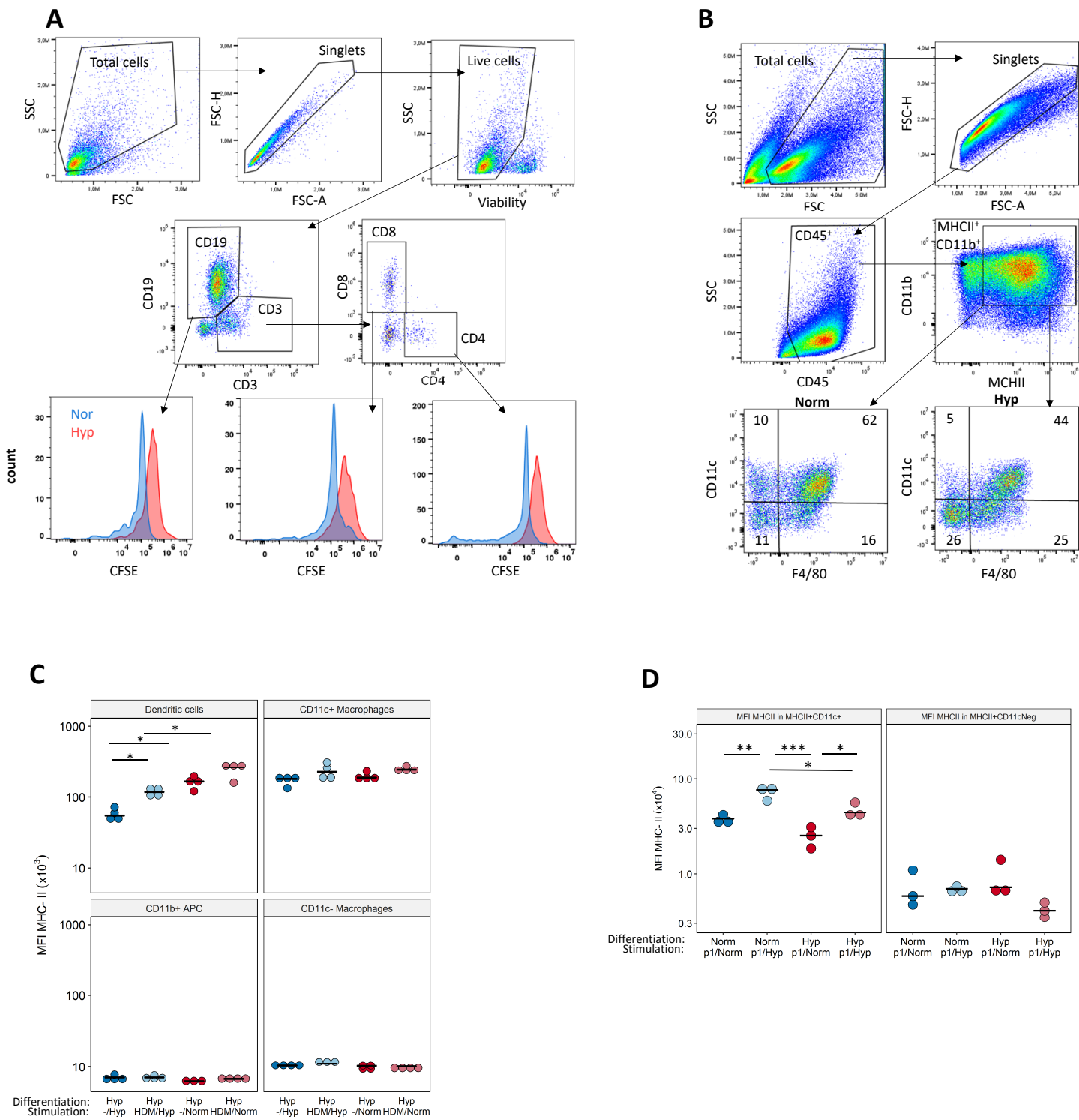

Supplement: Supplementary file 3 — Fig S3 [file ALL-77-870-s007.pdf]

Supplementary Figure E4

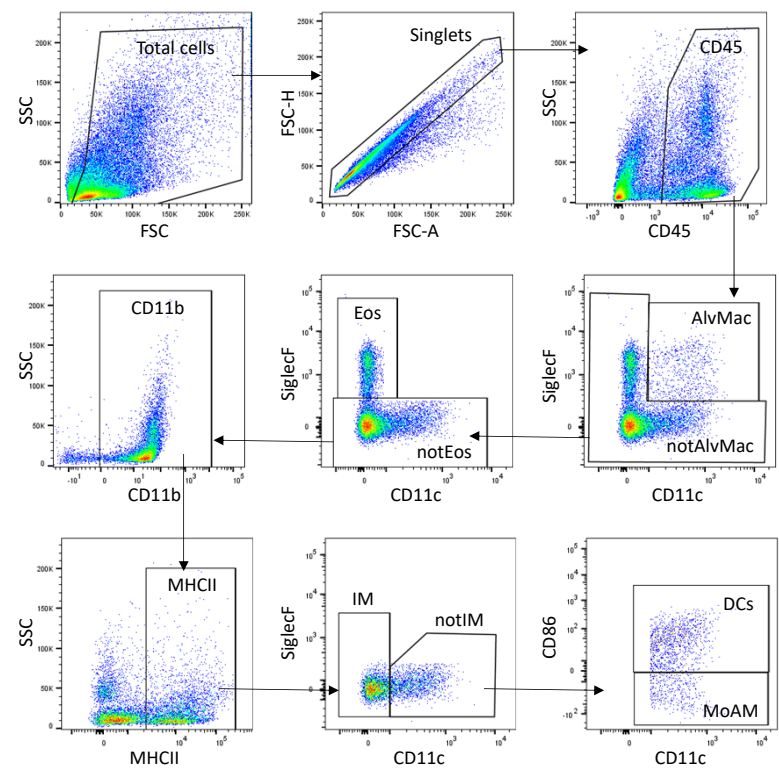

Supplement: Supplementary file 4 — Fig S4 [file ALL-77-870-s003.pdf]

Supplementary Figure E5

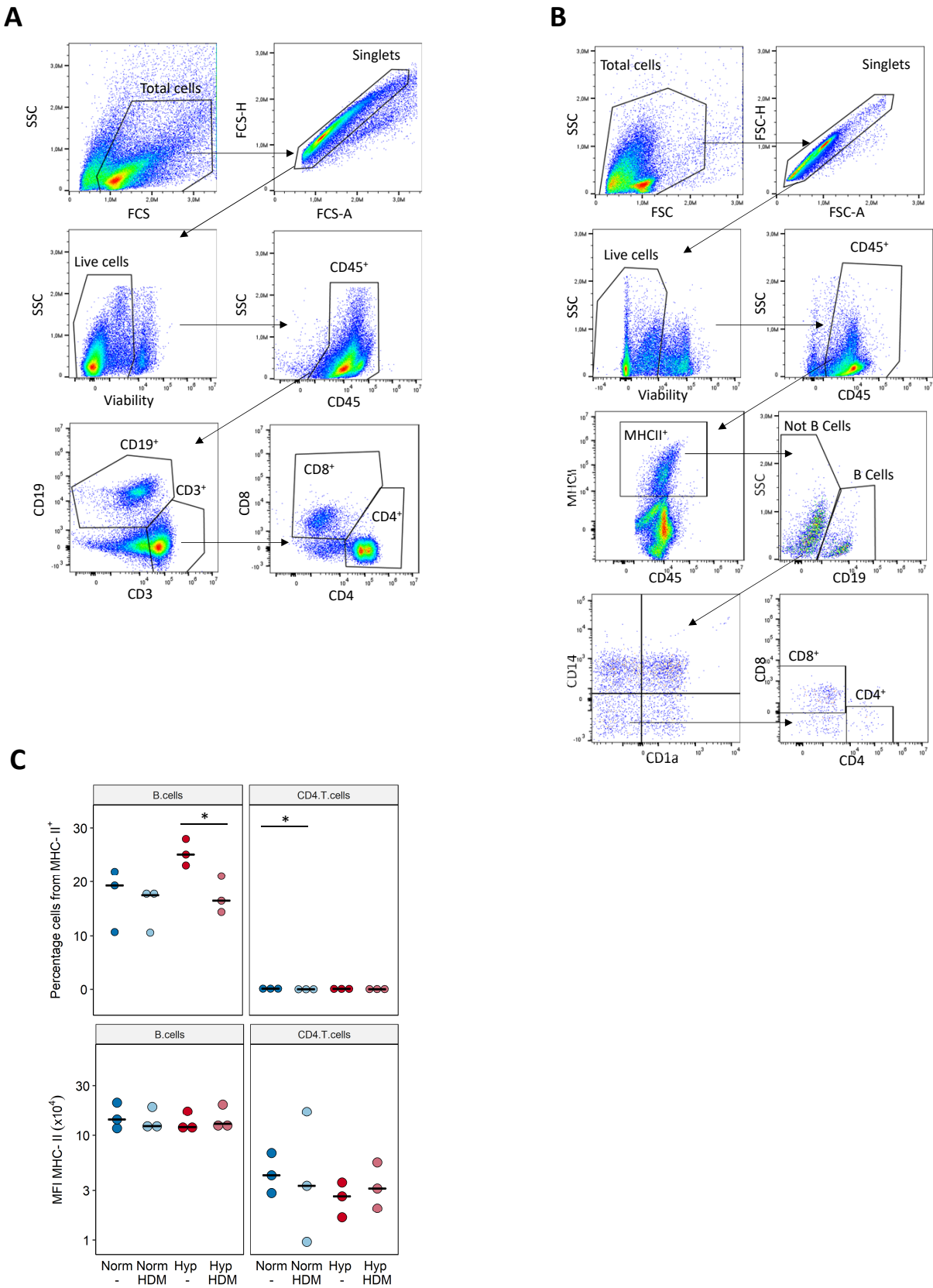

Supplement: Supplementary file 5 — Fig S5 [file ALL-77-870-s002.pdf]
