# Supplementary material for: Low oxygen levels decrease adaptive immune responses and ameliorate experimental asthma in mice
Source: Allergy. 2021 Aug 1;77(3):870–82. doi: 10.1111/all.15020 (PMC9290649; doi:10.1111/all.15020)
Supplement: Supplementary file 6 — Tab S1 [file ALL-77-870-s004.pdf]

Supplementary Table 1

Antigen	Label	Company	Clone	Isotype	Dilution
CD1a	BV 510	BDBioscience	PE	Mouse IgG1, κ	1:20
CD3	FITC	eBioscience	145-2C11	Hamster IgG	1:20
CD4	APC	Biolegend	GK1.5	Rat IgG2b, κ	1:100
CD8	PE	Biolegend	53-6.7	Rat IgG2a, κ	1:200
CD11b	V500	BDBioscience	M1/70	Rat IgG2b, κ	1:50
CD11c	ef450	eBioscience	N418	Hamster IgG	1:50
CD14	BV510	BDBioscience	MΦP9	Mouse IgG2b, κ	1:20
CD19	AF700	Biolegend	6D5	Rat IgG2a, κ	1:100
CD24	PerCP Cy5.5	BDBioscience	M1/69	Rat IgG2b, κ	1:500
CD25	APC-Cy7	Biolegend	PC61	Rat IgG1, λ	1:50
CD45	FITC/PerCP-Cy5.5	eBioscience	30-F11	Rat IgG2b, κ	1:200
CD64	AF647	BDBioscience	X54-5/7.1	Ms NOD/Lt IgG1, κ	1:20
CD86	PE-Cy5	eBioscience	GL1	Rat IgG2a, κ	1:50
Gr-1	PE-Cy7	Biolegend	RB6-8C5	Rat IgG2b, κ	1:800
MHC-II	APC-Cy7	Biolegend	M5/114.15.2	Rats IgG2b, κ	1:400
Siglec F	PE	BDBioscience	E50-2440	Rat IgG2a, κ	1:20
I-A/I-E	PerCP/Cy5.5	Biolegend	M5/114.15.2	Rat IgG2b, κ	1:50
CD3	BV510	Biolegend	145-2C11	Hamster IgG	1:50
CD19	PE-Cy7	eBioscience	eBio1D3	Rat IgG2a, κ	1:50
I-A/I-E	APC eFluor 780	eBioscience	M5/114.15.2	Rat IgG2a, κ	1:400
CD11b	SB600	eBioscience	M1/70	Rat IgG2a, κ	1:50
F4/80	eFlour 660	eBioscience	BM8	Rat IgG2a, κ	1:10
CD45	PerCP/Cy5.5	BDBioscience	HI30 (RUO)	Mouse IgG1, κ	1:100
CD4	PE-Cy7	eBioscience	SK3	Mouse IgG1, κ	1:20
CD8	SB600	eBioscience	RPA-T8	Mouse IgG1, κ	1:20
CD3	AF700	Biolegend	UCHT1	Mouse IgG1, κ	1:20
CD19	APC	Biolegend	HIB19	Mouse IgG1, κ	1:20
HLA-DR	APC-eFluor 780	eBioscience	LN3	Mouse IgG2b, κ	1:100
MHC-II	-	eBioscience	M5/114.15.2	Rat IgG2a, κ	1:5000
α-tubulin		Cell signalling	11H10	Rabbit IgG	1:5000
