# Supplementary material for: Low oxygen levels decrease adaptive immune responses and ameliorate experimental asthma in mice
Source: Allergy. 2021 Aug 1;77(3):870–82. doi: 10.1111/all.15020 (PMC9290649; doi:10.1111/all.15020)
Supplement: Supplementary file 7 — Tab S2 [file ALL-77-870-s009.pdf]

Supplementary Table 2

Gene	Species	Accession No.	Forward primer (5'-3')	Forward primer (3'-5')	Amplicon (bp)
IL-5	Mu	NM_010558.1	AAG CAA TGA GAC GAT GAG GCT	CCC CAC GGA CAG TTT GAT TCT	110
IL-10	Mu	NM_010548.2	AGG CGC TGT CAT CGA TTT CT	ATG GCC TTG TAG ACA CCT TGG	104
IL-13	Mu	NM_008355.3	GCC AAG ATC TGT GTC TCT CCC	CCA GGT CCA CAC TCC ATA CC	115
IL-17A	Mu	NM_010552.3	AGG ACG CGC AAA CAT GAG TC	GGA CAC GCT GAG CTT TGA GG	119
IFNg	Mu	NM_008337.3	CAG CAA CAG CAA GGC GAA AAA GG	TTT CCG CTT CCT GAG GCT GGA T	145
Muc5A	Mu	NM_010844.3	TGC TTC TGT CCT GAG GGT ATG	CAT GTG TTG GTG CAG TCA GTA G	137
Spdef	Mu	NM_013891.4	GAC GGA CGA CTC TTC TGA CA	CTG TTC GTG GTG CCA CAT CT	140
B2M	Mu	NM_009735.3	CGG CCT GTA TGC TAT CCA GAA AAC C	TGT GAG GCG GGT GGA ACT GTG	115
PBGD	Mu	NM_001110251	GCC AGA GAA AAGT GCC GTG GG	TCC GGA GGC GGG TGT TGA GG	115
