# Supplementary material for: Low oxygen levels decrease adaptive immune responses and ameliorate experimental asthma in mice
Source: Allergy. 2021 Aug 1;77(3):870–82. doi: 10.1111/all.15020 (PMC9290649; doi:10.1111/all.15020)
Supplement: Supplementary file 8 — Tab S3 [file ALL-77-870-s006.pdf]

Supplementary Table 3

Group	Allergies	Donor	Sex	Age	IgE Total	House Dust Mites	Grass/Grains (Timothy)	Tree Pollen (Birch)	Animals (Cat)	Milk protein	Peanut
Control	X	1	F	44	53.4	0.05	0.01	0	0	0.29	0
Control	X	5	M	27	11.4	0.23	0.07	0.01	0	0.01	0
Control	X	7	M	29	18.7	0.03	0.03	0.01	0.01	0.03	0.01
Control	X	9	M	25	14	0.03	0.04	0.31	0	0.04	0.02
Control	X	11	F	24	29.5	0.04	0	0	0.01	0.01	0
Control	X	13	M	25	9.64	0.04	0	0	0	0.01	0
Control	X	15	M	33	77.1	0.04	3.16	0.61	0.02	0.02	0.01
Control	X	17	M	31	61.6	0.14	0.01	0.01	0.09	0.11	0.13
Allergic	HDM/ Grass/ Pollen/ Animal Hair	4	F	43	396	1.18	2.78	25.1	2.57	0.19	0.35
Allergic	HDM/ Grass/ Pollen	6	M	28	689	37.6	72.9	0.05	0.67	0.5	1.9
Allergic	HDM/ Grass/ Pollen/ Animal Hair	8	M	36	287	21.2	0.69	0.33	0.35	0.06	0.53
Allergic	HDM/ Animal Hair	10	M	39	160	35.1	0.05	0.01	0.01	0.02	0.15
Allergic	HDM/ Animal Hair	12	M	28	1543	>100	22.4	0.06	0.69	0.49	1.15
Allergic	HDM/ Grass/ Animal Hair	14	F	32	164	24.1	10.5	0.02	1.16	0.03	0.03
Allergic	HDM/ Grass/ Pollen/ Animal Hair	16	F	27	126	2.22	17	3.47	0.67	0.04	0.23
