# Supplementary material for: Low oxygen levels decrease adaptive immune responses and ameliorate experimental asthma in mice
Source: Allergy. 2021 Aug 1;77(3):870–82. doi: 10.1111/all.15020 (PMC9290649; doi:10.1111/all.15020)
Supplement: Supplementary file 9 — Data S1 [file ALL-77-870-s001.docx]

## Data S1

## Online data supplement

Lower oxygen levels decrease adaptive immune responses and ameliorate experimental asthma in mice

Mathias Hochgerner, Eva Sturm, Diana Schnoegl, Grazyna Kwapiszewska, Horst Olschewski, Leigh M. Marsh

## Expanded Material and Methods

### Assessment of airway hyperresponsiveness (AHR)

Airway resistance and elastance was measured via FlexiVent (SciReq Inc., Montreal, PQ, Canada), as previously described (10, 11). In brief, 72h after the last dose of HDM, mice were anesthetised (150mg/kg ketamine plus 20mg/kg xylazine), intubated and mechanically ventilated at 150 breaths/min; tidal volume 10ml/kg; positive end expiratory pressure 2cmH_2_O. Airway resistance was analysed via changes in the dose-response-curves to methacholine (0, 1, 3, 10, 30 and 100mg/ml, Sigma Aldrich). For each dose, twelve snapshot perturbations were measured; between perturbation-sets, lung volume was normalized via two deep inflation manoeuvres.

### Lung immunohistochemistry and quantitative histology

3-µm lung sections were deparaffinized in xylene followed by decreasing concentrations of ethanol and then stained by periodic acid–Schiff staining (PAS) according to standard protocols. Stained sections were scanned using an Olympus VS120 slide scanning microscope at 40x (Olympus, Vienna, AT). The percentage of goblet cells and mucus volume was quantified using the NewCast software (Visiopharm, Hoersholm, Denmark) on automatically selected random regions. The Goblet and epithelial cells intersecting the basement airway membrane were counted and presented as percentage goblet cells; the volume of mucus was determined by point and intersection counting (10, 13).

### RNA isolation and real time PCR analysis

RNA-isolation from mouse lung was performed via the peqGOLD Total RNA Kit from Peqlab (Erlangen, Germany). Resulting total RNA was transcribed via the iScript™ cDNA Synthesis kit from Bio-Rad Laboratories (Hercules, CA, USA). Real-time PCR were taken via the QuantiFast® SYBR® Green PCR kit from Qiagen (Hilden, Germany) on a LightCycler® 480 System from Roche Applied Science (Vienna, Austria). PCR-conditions were as follows: 95°C for 5min, [95°C for 5sec, 60°C for 5sec, 72°C for 10sec] (45 repeats). Specificity of PCR was confirmed via gel electrophoresis of products and melting curve analysis. ΔCt values for the target genes was calculated as described previously described (12). In brief: ΔCt = meanCt reference genes (PBGD, B2M) – Ct target gene. See Table 1 for primer sequences.

### Preparation of Bronchoalveolar lavage fluid (BALF)

After sacrifice, BALF was obtained using 1 ml PBS containing 1 mM EDTA and protease inhibitor cocktail (ThermoFisher Scientific, Vienna, AT ). After centrifugation the supernatant was aliquoted and frozen at -80°C, and cell pellet was fixed with 1 ml 1% paraformaldehyde for 15 min on ice before being resuspended in MACS buffer (1x PBS, 2 mM EDTA, 0.5% BSA).

### Preparation of Lung tissue

Lungs were perfused with ice cold PBS via the right ventricle, the right lung was then paraffin embedded for histologic analysis. The left lobe was removed and either upper portion snap-frozen in liquid nitrogen, the lower left lobe was used for preparation of lung tissue homogenate. The lower left lobe was weighed and digested at 37°C for 40 min with Collagenase (200 ng/ml) and DNAse (200ng/ml) in RPM1 media (ThermoFisher Scientific). The cell preparation was then filtered through a 100 µm cell strainer and when necessary residual red blood cells were lysed with erythrolysis buffer (2.6 mM NH_4_Cl, 0.09 M KCO_3_, 0.6 M Titriplex III). Cells were counted and then fixed with 1% paraformaldehyde for 15 min on ice before being resuspended in MACS buffer.

## Supplementary Table 1

| **Antigen** | **Label** | **Company** | **Clone** | **Isotype** | **Dilution** |
| --- | --- | --- | --- | --- | --- |
| CD1a | BV 510 | BDBioscience | PE | Mouse IgG1, κ | 1:20 |
| CD3 | FITC | eBioscience | 145-2C11 | Hamster IgG | 1:20 |
| CD4 | APC | Biolegend | GK1.5 | Rat IgG2b, κ | 1:100 |
| CD8 | PE | Biolegend | 53-6.7 | Rat IgG2a, κ | 1:200 |
| CD11b | V500 | BDBioscience | M1/70 | Rat IgG2b, κ | 1:50 |
| CD11c | ef450 | eBioscience | N418 | Hamster IgG | 1:50 |
| CD14 | BV510 | BDBioscience | MΦP9 | Mouse IgG2b, κ | 1:20 |
| CD19 | AF700 | Biolegend | 6D5 | Rat IgG2a, κ | 1:100 |
| CD24 | PerCP Cy5.5 | BDBioscience | M1/69 | Rat IgG2b, κ | 1:500 |
| CD25 | APC-Cy7 | Biolegend | PC61 | Rat IgG1, λ | 1:50 |
| CD45 | FITC/PerCP-Cy5.5 | eBioscience | 30-F11 | Rat IgG2b, κ | 1:200 |
| CD64 | AF647 | BDBioscience | X54-5/7.1 | Ms NOD/Lt IgG1, κ | 1:20 |
| CD86 | PE-Cy5 | eBioscience | GL1 | Rat IgG2a, κ | 1:50 |
| Gr-1 | PE-Cy7 | Biolegend | RB6-8C5 | Rat IgG2b, κ | 1:800 |
| MHC-II | APC-Cy7 | Biolegend | M5/114.15.2 | Rats IgG2b, κ | 1:400 |
| Siglec F | PE | BDBioscience | E50-2440 | Rat IgG2a, κ | 1:20 |
| I-A/I-E | PerCP/Cy5.5 | Biolegend | M5/114.15.2 | Rat IgG2b, κ | 1:50 |
| CD3 | BV510 | Biolegend | 145-2C11 | Hamster IgG | 1:50 |
| CD19 | PE-Cy7 | eBioscience | eBio1D3 | Rat IgG2a, κ | 1:50 |
| I-A/I-E | APC eFluor 780 | eBioscience | M5/114.15.2 | Rat IgG2a, κ | 1:400 |
| CD11b | SB600 | eBioscience | M1/70 | Rat IgG2a, κ | 1:50 |
| F4/80 | eFlour 660 | eBioscience | BM8 | Rat IgG2a, κ | 1:10 |
| CD45 | PerCP/Cy5.5 | BDBioscience | HI30 (RUO) | Mouse IgG1, κ | 1:100 |
| CD4 | PE-Cy7 | eBioscience | SK3 | Mouse IgG1, κ | 1:20 |
| CD8 | SB600 | eBioscience | RPA-T8 | Mouse IgG1, κ | 1:20 |
| CD3 | AF700 | Biolegend | UCHT1 | Mouse IgG1, κ | 1:20 |
| CD19 | APC | Biolegend | HIB19 | Mouse IgG1, κ | 1:20 |
| HLA-DR | APC-eFluor 780 | eBioscience | LN3 | Mouse IgG2b, κ | 1:100 |
| MHC-II | - | eBioscience | M5/114.15.2 | Rat IgG2a, κ | 1:5000 |
| α-tubulin |  | Cell signalling | 11H10 | Rabbit IgG | 1:5000 |

## Supplementary Table 2

| **Gene** | **Species** | **Accession No.** | **Forward primer (5’-3’)** | **Forward primer (3’-5’)** | **Amplicon (bp)** |
| --- | --- | --- | --- | --- | --- |
| IL-5 | Mu | NM_010558.1 | AAG CAA TGA GAC GAT GAG GCT | CCC CAC GGA CAG TTT GAT TCT | 110 |
| IL-10 | Mu | NM_010548.2 | AGG CGC TGT CAT CGA TTT CT | ATG GCC TTG TAG ACA CCT TGG | 104 |
| IL-13 | Mu | NM_008355.3 | GCC AAG ATC TGT GTC TCT CCC | CCA GGT CCA CAC TCC ATA CC | 115 |
| IL-17A | Mu | NM_010552.3 | AGG ACG CGC AAA CAT GAG TC | GGA CAC GCT GAG CTT TGA GG | 119 |
| IFNg | Mu | NM_008337.3 | CAG CAA CAG CAA GGC GAA AAA GG | TTT CCG CTT CCT GAG GCT GGA T | 145 |
| Muc5A | Mu | NM_010844.3 | TGC TTC TGT CCT GAG GGT ATG | CAT GTG TTG GTG CAG TCA GTA G | 137 |
| Spdef | Mu | NM_013891.4 | GAC GGA CGA CTC TTC TGA CA | CTG TTC GTG GTG CCA CAT CT | 140 |
| B2M | Mu | NM_009735.3 | CGG CCT GTA TGC TAT CCA GAA AAC C | TGT GAG GCG GGT GGA ACT GTG | 115 |
| PBGD | Mu | NM_001110251 | GCC AGA GAA AAGT GCC GTG GG | TCC GGA GGC GGG TGT TGA GG | 115 |

## Supplementary Table 3

| **Group** | **Allergies** | **Donor** | **Sex** | **Age** | **IgE Total** | **House Dust Mites** | **Grass/Grains (Timothy)** | **Tree Pollen (Birch)** | **Animals (Cat)** | **Milk protein** | **Peanut** |
| --- | --- | --- | --- | --- | --- | --- | --- | --- | --- | --- | --- |
| Control | X | 1 | F | 44 | 53.4 | 0.05 | 0.01 | 0 | 0 | 0.29 | 0 |
| Control | X | 5 | M | 27 | 11.4 | 0.23 | 0.07 | 0.01 | 0 | 0.01 | 0 |
| Control | X | 7 | M | 29 | 18.7 | 0.03 | 0.03 | 0.01 | 0.01 | 0.03 | 0.01 |
| Control | X | 9 | M | 25 | 14 | 0.03 | 0.04 | 0.31 | 0 | 0.04 | 0.02 |
| Control | X | 11 | F | 24 | 29.5 | 0.04 | 0 | 0 | 0.01 | 0.01 | 0 |
| Control | X | 13 | M | 25 | 9.64 | 0.04 | 0 | 0 | 0 | 0.01 | 0 |
| Control | X | 15 | M | 33 | 77.1 | 0.04 | 3.16 | 0.61 | 0.02 | 0.02 | 0.01 |
| Control | X | 17 | M | 31 | 61.6 | 0.14 | 0.01 | 0.01 | 0.09 | 0.11 | 0.13 |
| Allergic | HDM/ Grass/ Pollen/ Animal Hair | 4 | F | 43 | 396 | 1.18 | 2.78 | 25.1 | 2.57 | 0.19 | 0.35 |
| Allergic | HDM/ Grass/ Pollen | 6 | M | 28 | 689 | 37.6 | 72.9 | 0.05 | 0.67 | 0.5 | 1.9 |
| Allergic | HDM/ Grass/ Pollen/ Animal Hair | 8 | M | 36 | 287 | 21.2 | 0.69 | 0.33 | 0.35 | 0.06 | 0.53 |
| Allergic | HDM/ Animal Hair | 10 | M | 39 | 160 | 35.1 | 0.05 | 0.01 | 0.01 | 0.02 | 0.15 |
| Allergic | HDM/ Animal Hair | 12 | M | 28 | 1543 | >100 | 22.4 | 0.06 | 0.69 | 0.49 | 1.15 |
| Allergic | HDM/ Grass/ Animal Hair | 14 | F | 32 | 164 | 24.1 | 10.5 | 0.02 | 1.16 | 0.03 | 0.03 |
| Allergic | HDM/ Grass/ Pollen/ Animal Hair | 16 | F | 27 | 126 | 2.22 | 17 | 3.47 | 0.67 | 0.04 | 0.23 |

## Supplemental Figure Legends

### Figure E1. Effects of hypoxia on pulmonary vascular remodelling

Mice were sensitised and challenged with a crude extract of HDM or PBS once a week over four weeks; after two weeks mice kept under room air (21% Oxygen) or reduced oxygen conditions (10% Oxygen) for an additional two weeks. Analysis was performed 72 hrs after the last challenge. A) Analysis of the Fulton Index (right ventricle (RV) / left ventricle (LV) + septum (S). B) Immunohistochemical staining against smooth muscle actin (purple) and von Willebrand Factor (vWF; brown) to determine extent of vascular remodelling. C) Quantification of (B). D) Mouse body weight at the end of experiment-*p<0.05, **p<0.01, ***p<0.001.

### Figure E2. FACS gating strategy to identify murine inflammatory cell populations in the lungs and bronchoalveolar lavage fluid in Figure 2.

### Figure E3. FACS gating strategy and MHC-II quantification for Figure 3.

A) Flow cytometry-gating strategy to determine T and B-cell proliferation as % CFSE-lo of parent population. B) Identification of Antigen presenting cells (APCs) differentiated from mouse bone marrow to BM-APCs for 6 days under Normoxia (Norm) or Hypoxia (Hyp). C) Mean fluorescent intensity (MFI) of MHC-II expression on BM-APCs generated under Hyp and stimulated under Norm or Hyp with or without 100 µg HDM. D) MHC-II MFI on CD11c^+^ and CD11c^-^ cells after stimulation with Ovalbumin-peptide 323-339 (p1) and co-culture with isolated CD4^+^ T-cells for 8 days. -*p<0.05, **p<0.01, ***p<0.001.

### Figure E4. FACS gating strategy to identify different antigen presenting cell populations in mouse lungs.

Flow cytometry of mouse lung single cell suspensions to identify alveolar macrophages (AlvMac), interstitial macrophages (IM), dendritic cells (DCs) and monocyte derived alveolar macrophages (MoAM) as analysed in Figure 4.

### Figure E5. FACS gating strategy to identify different antigen presenting cell (APCs) and T cell populations in isolated human peripheral blood mononuclear cells.

A) Identification of T and B-cells for the determination of cell proliferation as performed in Figure 5C. B) Identification of cells with potential MHC-II expression as quantified in Figure 5E-F. C) Analysis of MHCII-expressing cells in HDM-stimulated or unstimulated human PBMC cultures from allergic donors, Nor vs Hyp, B-cells and CD4+ T-cells, expressed as % of CD45+MHCII+ cells. -*p<0.05
